# Recurrent Takotsubo Cardiomyopathy Presenting With Anterior Wall ST-Elevation Myocardial Infarction

**DOI:** 10.7759/cureus.13458

**Published:** 2021-02-20

**Authors:** Maham A Waheed, Mazin Khalid, Arsalan Talib Hashmi, Jacob Shani, Bilal Malik

**Affiliations:** 1 Department of Internal Medicine, Maimonides Medical Center, Brooklyn, USA; 2 Department of Cardiology, Maimonides Medical Center, Brooklyn, USA

**Keywords:** st-elevation myocardial infarction (stemi), takutsobo, cardiomyopathy, recurrent

## Abstract

Takotsubo cardiomyopathy (TC) or stress cardiomyopathy with the presence of transient apical ballooning of the left ventricle in the absence of obstructive coronary artery disease. The recurrence of TC is extremely rare, with an annual recurrence risk of 1.5% and approximately 5% recurrence risk after six years. We present a case of a patient with a history of TC who presented with chest pain and ST-segment elevation in her electrocardiogram and was found to have normal coronaries and diagnosed with recurrent TC.

## Introduction

Takotsubo cardiomyopathy (TC) or stress cardiomyopathy was first described in 1990 as the presence of transient apical ballooning of the left ventricle in the absence of obstructive coronary artery disease [1]. It occurs predominantly in older women and is often preceded by an emotional or physical stress factor [2]. The classic variant or apical type of TC with transient apical ballooning and hyper contraction of basal left ventricle is usually the most common variant. However, atypical variants of TC with various segmental wall motion abnormalities, including basal, mid-ventricular, and right ventricular myocardium, have been reported [3,4]. Though the reasons for exact differences in regional susceptibility of segmental wall motion abnormalities in TC variants is unclear, it has been attributed to varying patterns of susceptibility to catecholamine excess in the ventricle [3]. The recurrence of TC is extremely rare, with an annual recurrence risk of 1.5% and approximately 5% recurrence risk after six years [5]. We present the case of a patient with a history of TC who presented with chest pain and ST-segment elevation in her electrocardiogram (ECG) and was found to have normal coronaries and diagnosed with recurrent TC.

## Case presentation

A 72-year-old woman with a past medical history of hypertension, diabetes mellitus, and TC eight years ago presented to the emergency room with severe retro-sternal crushing chest pain and associated nausea, vomiting, diaphoresis. On examination, she was afebrile, had a blood pressure of 90/60 mmHg, heart rate 66 bpm, respiratory rate 14 bpm, and was saturating 97% on room air. 

**Figure 1:**
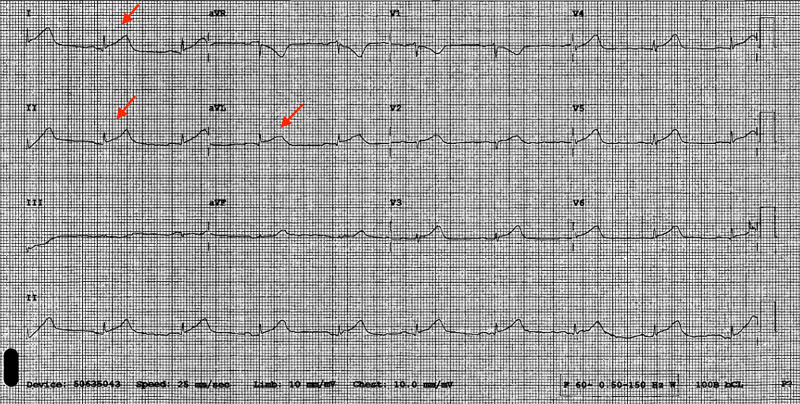
ECG at presentation showing ST-segment elevation leads I, II, aVL as shown with the red arrows. ECG: electrocardiogram.

Laboratory work upon admission revealed a WBC count of 6 K/UL, Hb 13.2 g/dL, platelet count 181 K/uL, serum troponin 0.38 ng/ml, serum creatine kinase-myocardial band (CK-MB) 4.2 ng/ml, serum myoglobin 157. An ECG performed at the bedside revealed ST elevations in leads I, II, and aVL (Figure 1).

**Figure 2:**
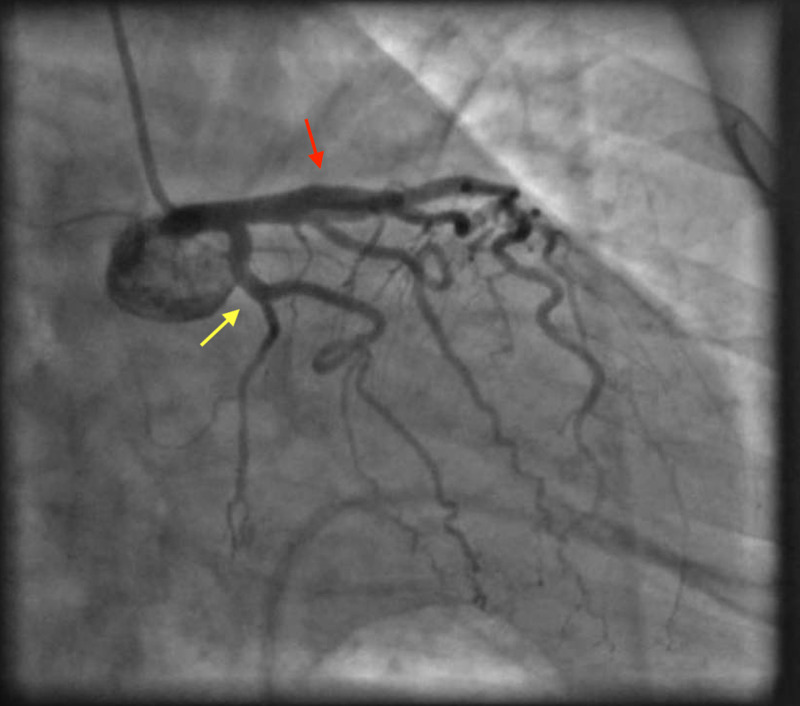
Coronary angiogram showing patent left anterior descending (red arrow) and left circumflex arteries (yellow arrow).

**Figure 3:**
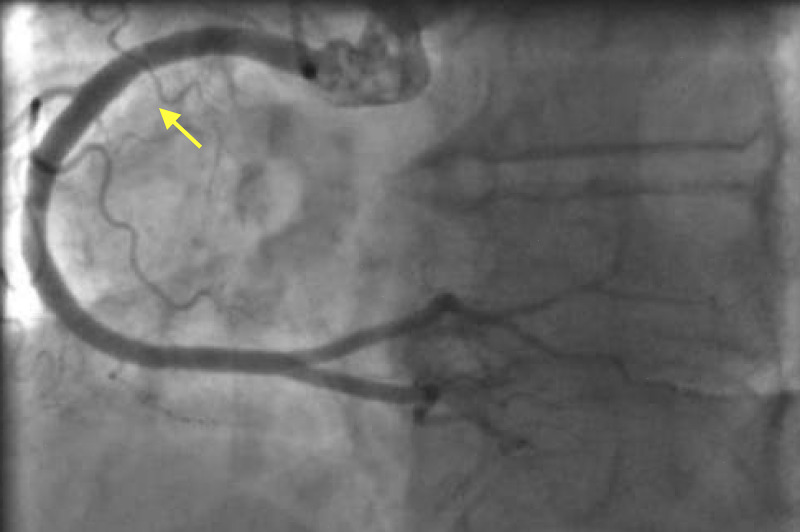
Right coronary angiogram showing patent right coronary artery.

**Figure 4:**
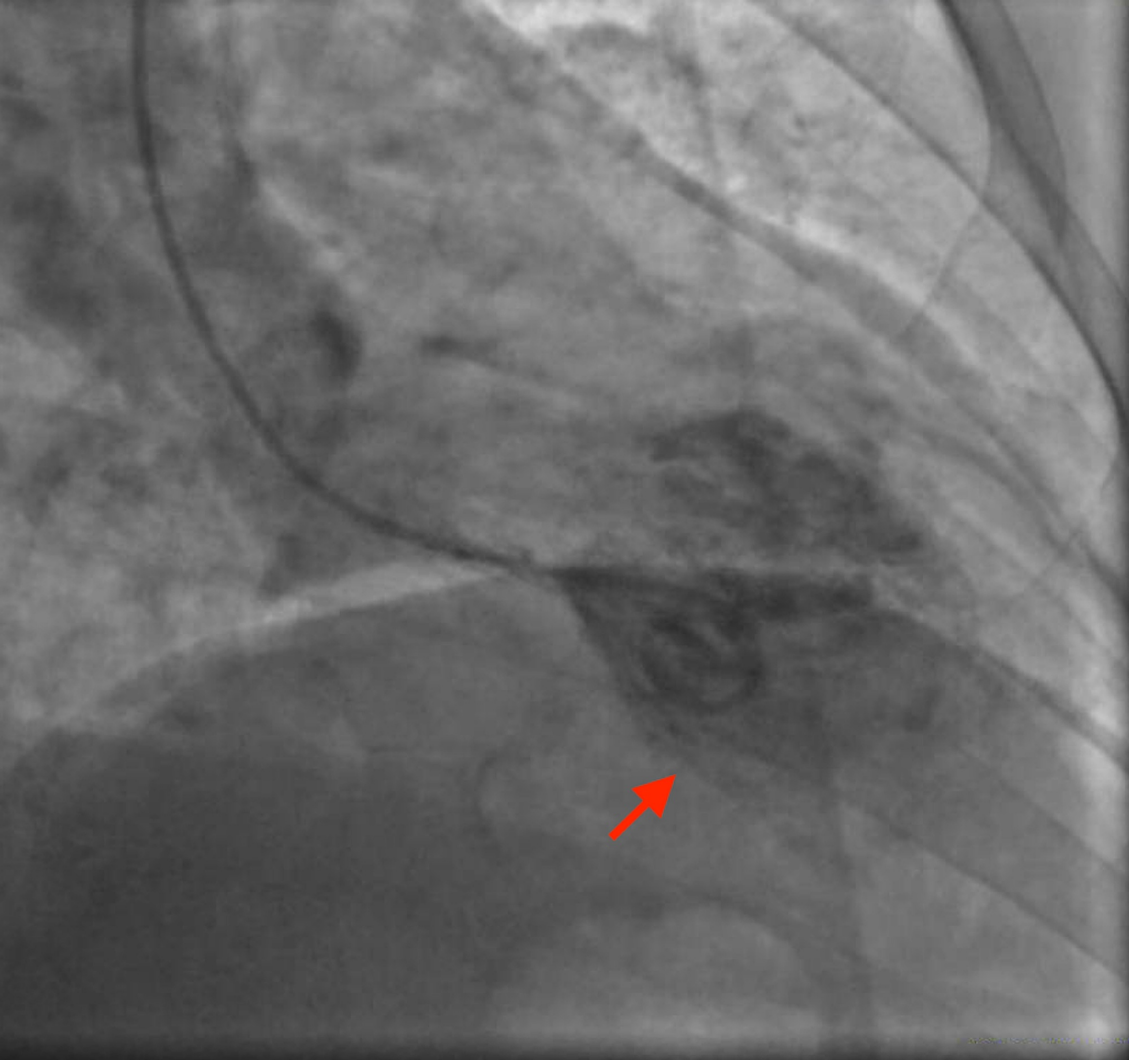
Left ventriculogram showing apical ballooning during systole as shown with the red arrow.

Given her risk profile, symptoms, and ECG findings, we performed an emergent coronary angiogram. She had normal coronaries, and her left ventriculogram showed apical ballooning consistent with TC (Figures 2, 3, 4).

**Figure 5:**
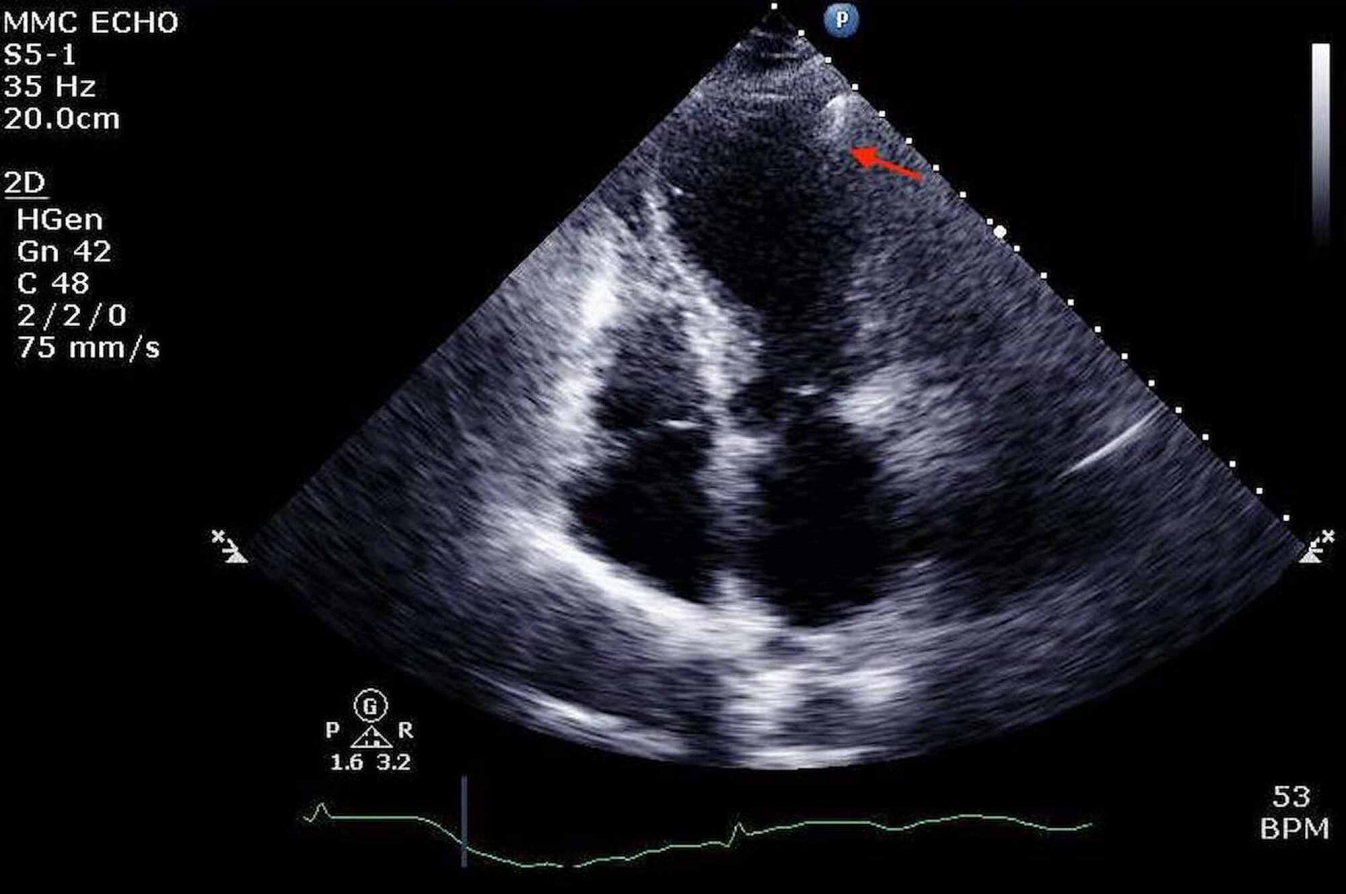
Transthoracic echocardiogram showing apical ballooning (red arrow). Apical four-chamber view showing systolic apical ballooning consistent with TC. TC: Takotsubo cardiomyopathy.

She was admitted to the coronary care unit for further management. After eight hours, the patient's serum troponin levels rose to 4.69 ng/ml. Further workup with transthoracic echocardiography (TTE) revealed severely decreased left ventricular systolic function with left ventricular ejection fracture (LVEF) of 21%-25%, akinetic left ventricular walls with multiple segmental abnormalities, and findings consistent with stress-induced cardiomyopathy. An impaired relaxation pattern of LV diastolic filling was also found (Figure 5) (Video 1).

**Video 1:** Transthoracic echocardiogram showing reduced wall motion in the apical segments with apical ballooning and reduced ejection fraction.

The patient later admitted to having a heated emotional argument with her daughter which is the likely predisposing event and it was similar to the setting of her previous TC event. She was started on metoprolol succinate, angiotensin-converting enzyme inhibitors, statin and observed with close hemodynamic monitoring. Over the week of her hospitalization, her EKG chawed resolved and the patient reported gradual improvement in her symptoms, and on day seven of admission, repeat TTE revealed improved left ventricular with EF of 35% and improvement of wall motion abnormalities.

## Discussion

TC is also known as "broken heart syndrome," often mimics acute coronary syndrome during acute presentation and is accompanied by reversible left ventricle apical ballooning in the absence of angiographic coronary stenosis; thus, making it a diagnosis of exclusion [2]. Usually, patients present with signs and symptoms concerning for acute myocardial infarction, with the most common symptom being precordial pain [6]. ECG findings are often consistent with ST-segment elevations or depression, T-wave changes, or a prolonged QT interval [7-9]. Further workup reveals elevated cardiac enzymes. TTE typically reveals apical ballooning of LV due to akinesia, hypokinesia, or dyskinesia of apical and middle segments of the LV and hyperkinesia of the basal segments [6,10,11]. The LVEF is decreased in most patients during the initial phase and improves gradually throughout the sub-acute phase, during which global and segmental systolic LV function begins to improve [6,12]. Cardiac Catheterization usually reveals normal coronary arteries without angiographic evidence of acute stenosis. Spontaneous coronary artery spasm on angiography has been reported to be in 1.4%-10% of patients, induced spasm in 4.5%-71%, and vasoconstriction in 21%-100% [6].

Though the exact pathogenesis involving the development of TC are unclear, multiple mechanisms have been proposed, such as coronary vasospasm, myocardial injury due to microvascular spasm, neurogenic stunned myocardium, multi-vessel epicardial spasm, as well as catecholamine excess mediated myocardial dysfunction [13-15]. The German Italian Stress Cardiomyopathy (GIEST) registry involving 749 patients with TC found TC recurrence to be 4% at a median follow-up of 27.6 months. Compared to the non-recurrence group, though baseline characteristics were the same, the recurrence group was found to have a significantly higher presence of arterial hypertension. Variable TC pattern was also common in up to 20% of cases within the recurrence group, and 46% of patients had a new stress trigger at the time of recurrence [16]. Treatment of patients admitted with TC should be individualized based on the patient's hemodynamic status. In hemodynamically stable patients, a combination of alpha and beta-blockade has been used to curb excess sympathetic activation. Beta-blockers have also been used to manage dynamic left ventricular outflow tract obstruction (LVOTO) in patients [17]. Although it has been proposed that ACE inhibitor therapy is more effective at decreasing the rate of TC recurrences than beta-blockers, a recent meta-analysis reveals that there is no evidence that drug therapy, inclusive of beta-blockers, aspirin, statins, ACE/ARB, prevents TC recurrence [5,18]. Another retrospective study evaluating pharmacologic therapy's efficacy with beta-blockers, ACE-inhibitors, calcium channel blockers, and aspirin is given until 30 days post-discharge in patients with TC also did not show any statistically significant difference in LVEF improvements [19]. On the contrary, ACE-inhibitors should be used with caution in such patients due to the posed risk of life-threatening hypotension. It has been found that approximately 10% of patients with TC also develop cardiogenic shock due to LVOTO. While intra-aortic balloon pump (IABP) is widely used to treat patients with cardiogenic shock, its use in patients with TC is not recommended as it can cause hemodynamic deterioration in the presence of LVOTO [20].

## Conclusions

Physicians should be mindful of the possibility of recurrent TC. In patients with history of prior TC, ischemic symptoms and ECG findings with normal angiography, recurrent TC should be suspected optimal medical therapy should be instated. The use of IABP should be avoided in hemodynamically unstable TC patients with LVOTO as it reduces the afterload increasing the LVOTO and worsening the hemodynamics.
